# Effect of mobile phone reminder messages on adherence of stent removal or exchange in patients with benign pancreaticobiliary diseases: a prospectively randomized, controlled study

**DOI:** 10.1186/s12876-016-0522-4

**Published:** 2016-08-26

**Authors:** Yong Gu, Limei Wang, Lina Zhao, Zhiguo Liu, Hui Luo, Qin Tao, Rongchun Zhang, Shuixiang He, Xiangping Wang, Rui Huang, Linhui Zhang, Yanglin Pan, Xuegang Guo

**Affiliations:** 1 Department of Gastroenterology, the first affiliated hospital of Xi’an Jiao Tong university, Xi’an, China; 2 Digestive System Department, Shaanxi Provincial Crops Hospital of Chinese People’s Armed Police Force, Xi’an, China; 3 Xijing Hospital of Digestive Diseases, Fourth Military Medical University, Xi’an, Shannxi China; 4 Department of Radiotherapy, Xijing Hospital, Xian, China

**Keywords:** Short message service, Adherence, Stent exchange, ERCP, Biliary stricture

## Abstract

**Background:**

Plastic and covered metal stents need to be removed or exchanged within appropriate time in case of undesirable complications. However, it is not uncommon that patients do not follow the recommendation for further stent management after Endoscopic Retrograde Cholangiopancreatography (ERCP). The effect of short message service (SMS) intervention monthly on the stent removal/exchange adherence in patients after ERCP is unknown at this time.

**Methods:**

A prospective, randomized controlled study was conducted. After receiving regular instructions, patients were randomly assigned to receive SMS reminding monthly (SMS group) for stent removal/exchange or not (control group). The primary outcome was stent removal/exchange adherence within appropriate time (4 months for plastic stent or 7 months for covered stent). Multivariate analysis was performed to assess factors associated with stent removal/exchange adherence within appropriate time. Intention-to-treat analysis was used.

**Results:**

A total of 48 patients were randomized, 23 to the SMS group and 25 to the control. Adherence to stent removal/exchange was reported in 78.2 % (18/23) of patients receiving the SMS intervention compared with 40 % (10/25) in the control group (RR 1.98, 95 % CI 1.16–3.31; *p* = 0 · 010). Among patients with plastic stent insertion, the median interval time from stent implantation to stent removal/exchange were 90 days in the SMS group and 136 days in the control respectively (HR 0.36, 95 % CI 0.16–0.84, *p* = 0.018). No difference was found between the two groups regarding late-stage stent-related complications. The rate of recurrent abdominal pain tended to be lower in SMS group without significant difference (8.7 vs 28 %, *p* = 0.144). Multivariate logistic regression analyses revealed that SMS reminding was the only factor associated with adherence of stent removal/exchange (OR 6.73, 95 % CI 1.64–27.54, *p* = 0.008).

**Conclusion:**

This first effectiveness trial demonstrated that SMS reminding monthly could significantly increase the patient adherence to stent removal/exchange after ERCP.

**Trial registration:**

The study was respectively registered on July 10 in 2016 at ClinicalTrials.gov (NCT02831127).

## Background

Endoscopic implantation of plastic or covered metal stents is widely used in a variety of benign pancreaticobiliary diseases, including duct stricture, large or difficult stones, bile or pancreatic duct leak, etc. [1–4]. There are some complications after stent insertion, such as stent occlusion, proximal or distal migration, secondary duct injury and even the failure of stent removal [5–8]. For plastic stents, occlusion is the main disadvantage, limiting their patency to around 3 months. For fully covered metal stents, stent migration, occlusion and even the failure of stent removal may happen after long-term implantation [8, 9]. The longer the stents areplaced, more likely the complications may happen.

Although the optimal time of stent placement has not been well established, it has been recommended that plastic stent should be removed/exchanged within 3–4 months and covered metal stent be removed within 6 months [10]. However, it is not uncommon that patients with stent implantation do not follow the recommendation of further stent management [11]. With the stents left in biliary or pancreatic duct for a long-term period, stone formation, acute duct inflammation and even chronic pancreatitis and secondary sclerosing cholangitis can happen. Occasionally, breakage of the stent can be also found [12]. Some patients in this situation may need emergent endoscopic management or even surgery. In addition, endoscopic management may thus be technically challenging, and the treatment cost can be increased.

Many methods have been used to improve the adherence of patients in medical service [13–15]. With the advance of mobile technology and popular use of mobile phones, it is believed that the patient-centered outcome (e.g. suppressed viral loads due to antivirus treatment) can be improved by mobile telecommunication with the timely support of a patient by a health professional [13]. Here we hypothesize that mobile technology, reminding the patients the necessity of stent management in time by short message service (SMS), may increase the patient adherence. The purpose of this prospectively randomized, controlled study is to evaluate the effect of SMS intervention monthly on the stent removal/exchange adherence in patients with benign pancreaticobiliary diseases after ERCP.

## Methods

### Patients

This is a prospective, randomized, controlled study with consecutive patients with benign pancreaticobiliary diseases undergoing endoscopic stent insertion at Endoscopy Center of Xijing Hospital of Digestive Diseases in China. The study protocol and informed consent form were approved by Institutional Review Board of Xijing Hospital (protocol number: 20160707–1). The study was respectively registered on July 10 in 2016 at ClinicalTrials.gov (NCT02831127). The informed consent was obtained from all patients. Patients more than 18 years old with plastic or covered stent implantation for the drainage of bile or pancreatic juice were eligible for participation in the study. Patients should be able to communicate via SMS by mobile phones of themselves or relatives living together. Exclusion criteria included: 1. primary or secondary sclerosing cholangitis (PSC), 2. malignant or suspected malignant stricture of biliary or pancreatic duct, 3.implantation of pancreatic duct (PD) stent for prevention of post-ERCP pancreatitis, 4.expected survival time less than 6 months, 5. plan of surgery within 6 months, 6. pregnant or lactating women, 7. patients who could not give informed consent.

Written informed consent was obtained from all the patients. Patients were randomized (1:1) to either the SMS intervention (SMS group) or standard care (control group) after stent insertion by opening an opaque and sealed envelope. The envelopes were randomized by using computer-generated random numbers generated by one of the investigators (HR) who kept the randomization key under lock until the inclusion of the last patient. At least two telephone numbers of all patients or their relatives living together were recorded in case of failed connection later. In the beginning of the enrollment, all patients were instructed not to tell doctors, nurses and investigators whether they received SMS reminding or not. The investigator (ZLN) performing data analysis was blinded to the allocation until the final analysis was finished.

### Endoscopic treatment

The diagnosis of all the patients was primarily based on symptoms, surgical history, chemical test and imaging modalities (contrast-enhanced CT or ultrasound). All patients underwent MRCP for determination of etiology and the site of stricture. Only the patients with benign stricture of CBD or PD were considered eligible for this study. During ERCP, tissue samples were obtained with brush and/or forceps to confirm the benign nature of the stricture when clinically indicated. Single or multiple plastic stents (8.5Fr, Advanix, Boston Scientific, Natick, MA) or a fully covered self-expandable metal stent (FCSEMS) (Wallflex, Boston Scientific, Natick, MA) was inserted across the site of obstruction. The length of the stent varied depending on the anatomic location of the stricture. No covered metal stent was placed in PD. The number and type of the stents was determined based on the characteristics of stricture or diseases, which was determined at the discretion of the attending endoscopists.

### Intervention

After stent implantation, all patients received oral and written instructions about further management. If single or multiple plastic stents were inserted, patients were informed to come back to the hospital at 3 months for stent removal/exchange; if FCSEMS was inserted, they were informed to come back to the hospital at 6 months after ERCP. Patients in SMS group received additional reminding by SMS messages from an investigator (TQ) blinded to further clinical data collection. Each month after stent implantation, the investigator sent a text message by SMS to inform patients the necessity of regular stent removal/exchange and the disadvantage of delayed management, and to remind them the appropriate date to come back to the hospital for stent management. Patients were requested to respond by SMS and were encouraged to contact the investigator if they had any questions about stent management. Patients in control group were not contacted after ERCP. At the end of the study, all the patients who did not come back to the hospital were called and informed again to return for further stent management. Follow-up was at least 6 months for all patients.

### Outcome measurement

The primary outcome was stent removal/exchange adherence within appropriate time (4 months for plastic stent or 7 months for covered stent). Secondary outcomes were stent-related complications, including cholangitis, stent migration and abdominal pain.

### Statistical analysis

At the beginning of the study, a sample size calculation was performed. Based on our previous experience, only 1/3 of patients in common practice will readmit for stent removal/exchange within appropriate time. The adherence in SMS group was estimated to be 80 %. To detect the difference with a significance level (α) of .05 and a power of 80 % with a 2-tailed test, we calculated that at least 42 patients were needed. However, about 10 % of patients might be lost during follow up. Thus, we estimated that totally 48 patients would be enough for the detection of a significant difference in the primary outcome.

Intention-to-treat (ITT) analysis was used to assess primary outcome from all evaluable patients. Relative risk (RR) was reported for adherence, with an RR more than 1 suggesting better outcome for SMS intervention group. Since only a small group of patients would be included, categorical variables, such as adherence rate of stent exchange/removal and complication rates, were analyzed using Fisher’s exact test. Continuous variables were expressed as means with standard deviations and analyzed with student’s t-test. Cumulative proportion of patients readmitting with plastic stent implanted during follow up was determined by the Kaplan-Meier method, and the difference was assessed using the log-rank test.

To assess factors associated with stent removal/exchange adherence, multivariate logistic analysis was performed using variables with *p* values of <0.1 in the univariate logistic analysis. Forward stepwise method was used in the multivariate model. Analyses were performed with SPSS software version 19.0 for Windows (SPSS Inc, IBM Company). A *p* value <0.05 was considered statistically significant.

## Results

**Fig. 1:**
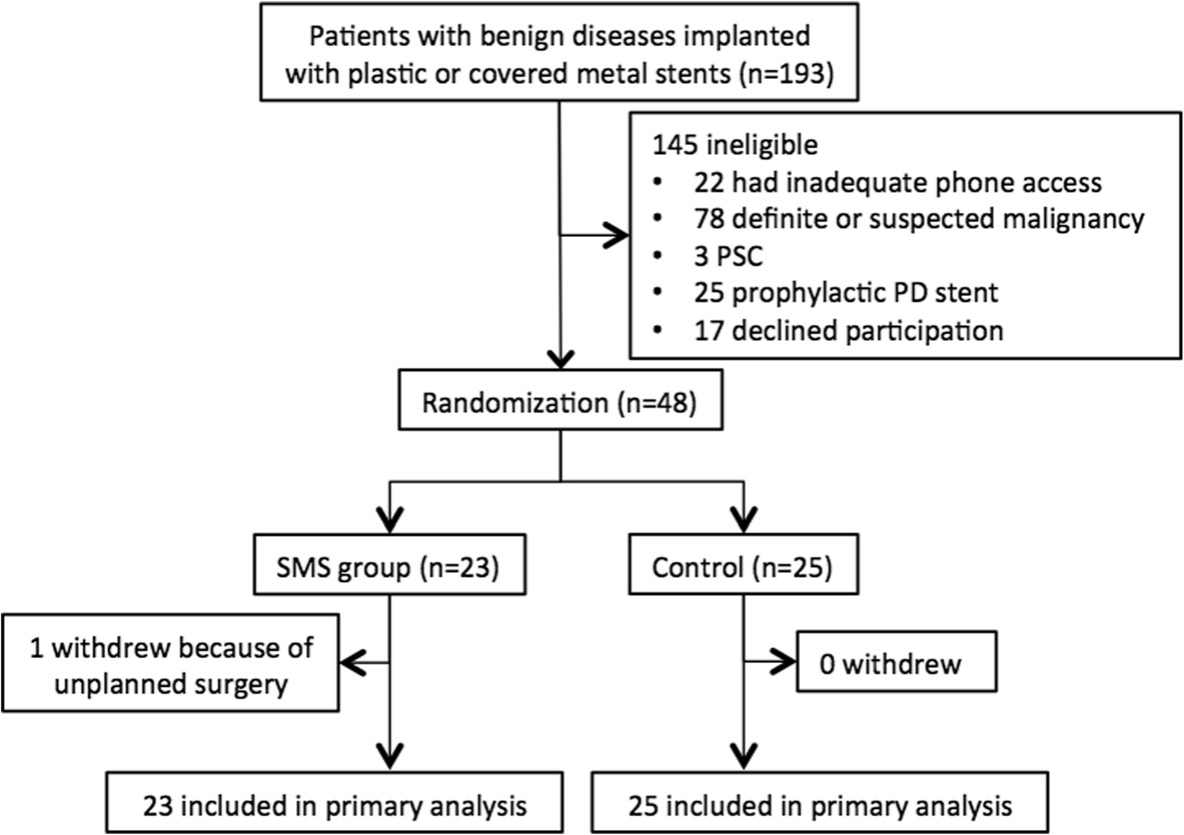
Flowchart of the study

From Feb in 2012 to Oct in 2013, 193 consecutive patients were enrolled. After screening, 145 patients were excluded, including 22 with inadequate phone access, 78 with definite or suspected malignancy, 3 with PSC, 25 with prophylactic PD stent implanted and 17 with declined participation. Finally, 48 patients were randomly assigned to the SMS group (*n* = 23) or to control group (*n* = 25). After randomization, all the patients in SMS group responded by SMS or phone call. However, 1 subject with distal stricture of CBD in SMS group underwent unplanned surgery because of pancreatic cancer. The subject flow is detailed in Fig. 1. All baseline characteristics but alkaline phosphatase (336.5 ± 324.2 U/L in SMS group vs. 125.8 ± 76.2 U/L in control, *p* = 0.003) between the two groups were well balanced (Table 1).

**Table 1:** Baseline of the characteristics of patients

	SMS group (*n* = 23)	Control (*n* = 25)	*P* value
Age	54.4 ± 15.0	52.2 ± 19.5	0.672
Male (%)	14 (60.9 %)	11 (44 %)	0.265
Smoking	7	3	0.162
Drinking	4	6	0.727
Education			1.000
Elementary school or less	5	6	
High school or higher	18	19	
Payment			0.610
By insurance	22	22	
By self	1	3	
Previous Stenting			0.511
Yes	7	5	
No	16	20	
Previous surgery			0.818
Cholecystectomy	8	7	
Liver transplantation	0	1	
Other	3	2	
Main symptom			0.467
Jaundice	7	4	
Fever	1	2	
Abdominal pain	13	16	
Chemical test before ERCP			
White blood cell (×10^9^)	5.8 ± 1.3	5.6 ± 2.8	0.714
Total bilirubin (mg/dL)	60.6 ± 93.3	30.0 ± 65.9	0.193
Alkaline phosphatase (U/L)	336.5 ± 324.2	125.8 ± 76.2	0.003
Stricture site			0.849
Proximal CBD	6	7	
Distal CBD	11	10	
PD	6	8	
Reason for stenting			0.501
Biliary benign stricture	10	12	
Pancreatic benign stricture	10	8	
Other	3	5	
Stent type			0.719
Plastic stent (average number of stents)	18 (1.39)	21 (1.43)	
FCSEMS	5	4	
ERCP complication			1.000
Pancreatitis	1	1	
Biliary infection	2	1	

*CBD* common bile duct, *PD* pancreatic duct, *FCSEMS* fully covered self-expanded metal stent

**Fig. 2:**
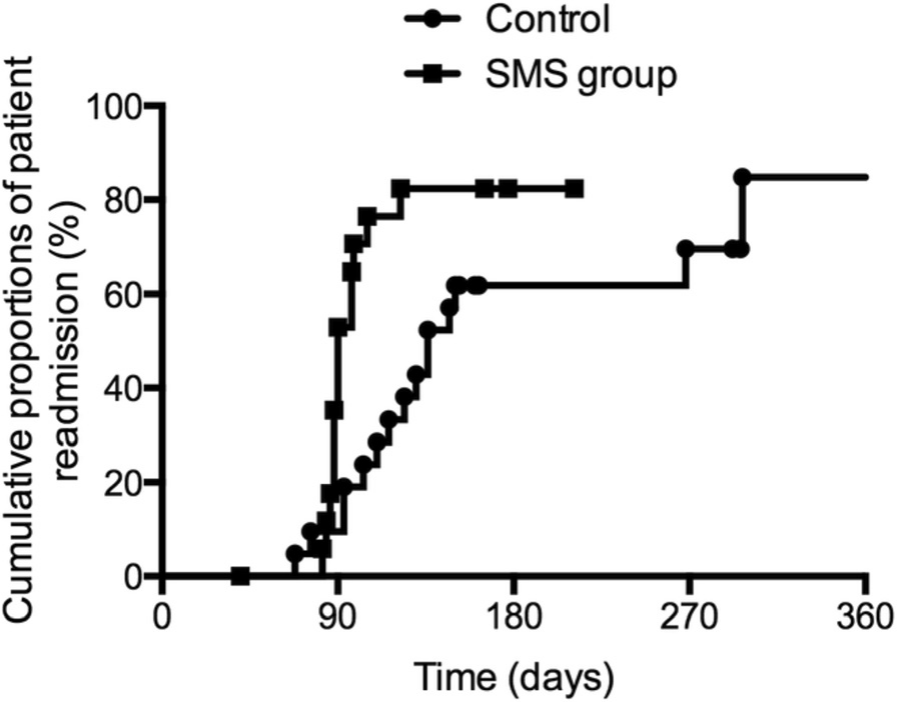
Kaplan-Meier survival analysis of proportions of patients with plastic stent implanted undergoing stent removal/exchange later in SMS group (*n* = 17) and control (*n* = 21). *p* = 0.018 by log-rank test

In ITT analysis, adherence to stent removal/exchange was reported in 78.2 % (18/23) of patients receiving the SMS intervention compared with 40 % (10/25) in the control group (relative risk [RR] 1.98, 95 % CI 1.16–3.31; *p* = 0 · 010) (Table 2). Among patients undergoing insertion of plastic stent (*n* = 39), adherence to stent removal/exchange was 77.8 % in SMS group and 33.3 % in control (*p* = 0.010). The cumulative proportions of patients coming back to the hospital during follow up are shown in Fig. 2. The mean interval time between stent implantation and stent removal/exchange was 90 days in SMS group and 136 days in the control group respectively (hazard ratio [HR] 0.36, 95 % CI 0.16–0.84, *p* = 0.018).As shown in Table 2, no difference was found regarding FCSEMS removal adherence between the two groups (80 vs. 75 %, *p* = 1.000). There were also no differences between the two groups with regard to stent-related complications, such as cholangitis (9 vs 8 %, *p* = 1.000), stent migration (13 vs. 8 %, *p* = 0.653) and recurrent abdominal pain (9 vs. 28 %, *p* = 0.144). However, the rate of recurrent abdominal pain tended to be lower in SMS group (8.7 vs 28 %, *p* = 0.144).

**Table 2:** Outcomes of SMS reminding compared with standard care

	SMS group (*n* = 23)	Control (*n* = 25)	*P* value
Stent removal/exchange adherence, n (%)	18/23 (78 %)	10/25 (40 %)	0.010
Plastic stent (<4 month)	14/18 (78 %)	7/21 (33 %)	0.010
FCSEMS (<7 month)	4/5 (80 %)	3/4 (75 %)	1.000
Stent-related complications, n (%)			
Cholangitis	2 (9 %)	2 (8 %)	1.000
Stent migration	3 (13 %)	2 (8 %)	0.653
Recurrent pain	2 (9 %)	7 (28 %)	0.144

FCSEMS, fully covered self-expanded metal stent

Multivariate logistic regression analyses were performed to identify any significant factors for stent removal or exchange adherence. The factors analyzed were age, gender, history of surgery, education level, pre-ERCP total bilirubin level, location of stenosis, stent type, stent number, reasons for stenting, post-ERCP complications and SMS reminding or not. As shown in Table 3, only SMS reminding were significantly associated with adherence of stent removal/exchange (OR 6.73, 95 % CI 1.64–27.54, *p* = 0.008).

**Table 3:** Multivariate logistic regression analysis of the association between patient characteristics and stent removal/exchange adherence

Variable		Adherence		*p* value
		OR	95 % CI	
SMS reminding	No	1		
	Yes	6.73	1.64–27.54	0.008
Surgery history	No	1		
	Yes	3.20	0.74–13.80	0.119
Stent type	Plastic	1		
	Metal	2.34	0.33–16.71	0.398
Stent number	Single	1		
	Multiple	2.10	0.51–8.73	0.306

Among patients coming back to the hospital finally (19 in SMS group vs 18 in the control, *p* = 0.297), 11 in the SMS group and 9 in the control group underwent plastic stent exchange (*p* = 0.746). The remaining patients in both groups needed no further management after stent removal and clearance of biliary or pancreatic duct.

## Discussion

Plastic stents and covered metal stent are commonly used for the drainage and relief of benign stricture of biliary and pancreatic ducts [1–4]. It is suggested that these stents should be removed or exchanged within 3–6 months to prevent late complications [7, 8]. Although patients are usually instructed the details of further stent management, some of them may be not compliant with the recommendation. The reasons may include: 1, the unawareness of the necessity of regular stent removal/exchange; 2, the unawareness of the possible complications of delayed stent management; 3, forgetting the appropriate date to come back to the hospital for stent management; 4, financial consideration. Here we found that SMS reminding monthly could significantly increase the patient adherence to stent removal/exchange. This is, to our knowledge, the first effectiveness trial assessing the ability of a mobile health technology intervention to influence the stent removal/exchange adherence.

Patients’ forgetfulness is considered one of the main reasons for missed appointments. There are many modes of communicating reminders for appointments to patients, such as face-to-face communication, postal messages, phone calls and SMS [16]. The later represent a convenient, less time-consuming and inexpensive delivery medium for improving the adherence of healthcare appointments. Studies that compare the outcomes of SMS reminding versus other methods for the patients with removable stents is of interest.

With the better adherence to stent removal or exchange, it could be expected that the stent-related complications due to long-term placement of plastic or cover metal stents might be reduced [7, 8, 17, 18]. However, the late-stage complications between the two groups in this study were not significantly different, although the rate of recurrent abdominal pain tended to be lower after SMS reminding. The reason may be due to small numbers of patients enrolled in each subgroup. The power of the study may be insufficient to detect the differences of stent-related complications and identify more predictive factors related to stent removal/exchange adherence.

There are some other limitations of this study. Firstly, the follow up time of this study is relatively short. It has been recommended that multiple plastic stents should be placed and exchanged for at least one year for long-term stricture of biliary stricture [19]. With better adherence to plastic stent exchange, it will be interesting to further evaluate the long-term resolution rate of biliary stricture after SMS reminding. Secondly, although patients with plastic stents in SMS group had better adherence to stent removal or exchange, no difference was found regarding the adherence to covered metal stent management. It is necessary to enroll more patients with covered metal stent to investigate whether they will be also benefit from SMS reminding. Thirdly, although number of patients undergoing placement of covered metal stent was similar between the two group, whether patients received metal stent were determined at the discretion of the attending endoscopists. The possible bias of patient selection may have impacts on the adherence in metal group. Last but not the least, the present study was performed in one tertiary center in a less developed area in China. The adherence rate without interference seems to be quite low (40 %). The beneficial effect of SMS on adherence of stent removal/exchange needs to be further investigated in other settings, especially in centers with higher adherence of stent removal/exchange.

## Conclusions

In conclusion, our study demonstrated that SMS reminding could improve the patient adherence to stent removal/exchange within appropriate time for the first time. SMS reminding could shorten the mean interval time between stent implantation and stent removal/exchange. Patients with stent implantation might be benefit from SMS reminding strategy.

## Abbreviation

CI: Confident interval
CT: Computed tomography
ERCP: Endoscopic retrograde cholangiopancreatography
FCSEMS: Fully covered self-expandable metal stent.
HR: hazard ratio
ITT: Intention-to-treat
MRCP: Magnetic resonance cholangiopancreatography
OR: Odd ratio
PD: Pancreatic duct
PSC: Primary sclerosing cholangitis
RR: Relative risk
SMS: Short message service
